# 1,3-Bis(2,6-diisopropyl­phen­yl)-1*H*-imidazol-3-ium chloride dichloro­methane disolvate

**DOI:** 10.1107/S1600536812022234

**Published:** 2012-05-23

**Authors:** Matthias Berger, Norbert Auner, Michael Bolte

**Affiliations:** a Institut für Anorganische und Analytische Chemie, Goethe-Universität Frankfurt, Max-von-Laue-Strasse 7, 60438 Frankfurt am Main, Germany

## Abstract

In the title compound, C_27_H_37_N_2_^+^·Cl^−^·2CH_2_Cl_2_, the cation and the anion are each located on a crystallographic mirror plane. Both of the dichloro­methane solvent mol­ecules show a disorder across a mirror plane over two equally occupied positions. Additionally, one isopropyl group is also disordered. In the crystal, the cations are connected to the chloride ions *via* C—H⋯Cl hydrogen bonds.

## Related literature

For the preparation of imidazolium salts, see: Arduengo *et al.* (1995 ▶, 1999 ▶); Hinter­mann (2007 ▶). For structures with the same cation but different anions, see: Stasch *et al.* (2004 ▶); Blue *et al.* (2006 ▶); Berger *et al.* (2012 ▶). For compounds with the 1,3-bis-(2,6-diisopropyl­phen­yl)imidazolium unit, see: Ikhile *et al.* (2010 ▶); Giffin *et al.* (2010 ▶).
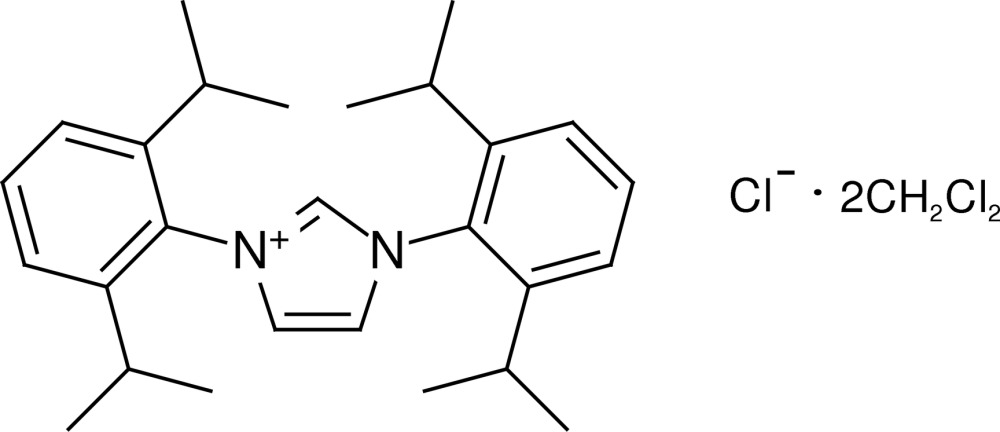


## Experimental

### 

#### Crystal data

C_27_H_37_N_2_^+^·Cl^−^·2CH_2_Cl_2_
*M* *_r_* = 594.89
Monoclinic, 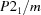

*a* = 9.1117 (4) Å
*b* = 16.4990 (8) Å
*c* = 10.8875 (6) Å
β = 101.068 (4)°
*V* = 1606.32 (14) Å^3^
*Z* = 2
Mo *K*α radiation
μ = 0.47 mm^−1^
*T* = 173 K
0.32 × 0.29 × 0.14 mm

#### Data collection

Stoe IPDS II two-circle diffractometer
Absorption correction: multi-scan (*MULABS*; Spek, 2009 ▶; Blessing, 1995 ▶) *T*_min_ = 0.864, *T*_max_ = 0.937
17793 measured reflections
2934 independent reflections
2648 reflections with *I* > 2σ(*I*)
*R*_int_ = 0.067

#### Refinement

*R*[*F* ^2^ > 2σ(*F* ^2^)] = 0.075
*wR*(*F* ^2^) = 0.184
*S* = 1.04
2934 reflections
185 parameters
6 restraints
H-atom parameters constrained
Δρ_max_ = 1.36 e Å^−3^
Δρ_min_ = −1.60 e Å^−3^

### 

Data collection: *X-AREA* (Stoe & Cie, 2001 ▶); cell refinement: *X-AREA*; data reduction: *X-AREA*; program(s) used to solve structure: *SHELXS97* (Sheldrick, 2008 ▶); program(s) used to refine structure: *SHELXL97* (Sheldrick, 2008 ▶); molecular graphics: *XP* in *SHELXTL* (Sheldrick, 2008 ▶); software used to prepare material for publication: *SHELXL97*.

## Supplementary Material

Crystal structure: contains datablock(s) I, global. DOI: 10.1107/S1600536812022234/ng5271sup1.cif

Structure factors: contains datablock(s) I. DOI: 10.1107/S1600536812022234/ng5271Isup2.hkl

Supplementary material file. DOI: 10.1107/S1600536812022234/ng5271Isup3.cml

Additional supplementary materials:  crystallographic information; 3D view; checkCIF report

## Figures and Tables

**Table 1:** Hydrogen-bond geometry (Å, °)

*D*—H⋯*A*	*D*—H	H⋯*A*	*D*⋯*A*	*D*—H⋯*A*
C1—H1⋯Cl1	0.95	2.50	3.447 (4)	176

**Fig. 1.**
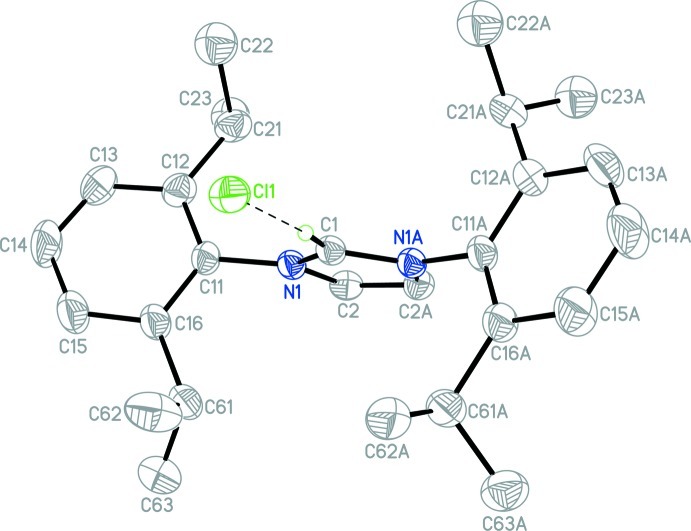
A perspective view of the title compound, showing the atom-numbering scheme. Displacement ellipsoids are drawn at the 50% probability level. Hydrogen bonds are shown as dashed lines. The minor occupied methyl groups, H atoms not involved in hydrogen bonding and dichloromethane molecules are omitted for clarity. Atoms labelled with suffix A were generated by the symmetry operator x, -y + 3/2, z.

## supplementary crystallographic information

### Comment

Imidazolium salts are precursors for the synthesis of N-heterocyclic carbenes (NHC) and can be prepared according to Arduengo *et al.* (1995, 1999) and Hintermann (2007). Deprotonation by strong bases gives the free stable NHC, which is widely used as ligands.

The title compound crystallizes with discrete cations, anions and solvent dichloromethane molecules. Both cations and anions are located on a crystallographic mirror plane. Both dichloromethane molecules show a disorder across a mirror plane over two equally occupied positions. Additionally, one isopropyl group is disordered as well. The Cl anions are connnected to the cations *via* C—H···Cl hydrogen bonds. Structures with the same cation, but with different anions and solvent molecules, have been determined by Stasch *et al.* (2004), Blue *et al.* (2006) and Berger *et al.* (2012). For the compounds with 1,3-bis-(2,6-diisopropylphenyl)imidazolium unit, see: Ikhile *et al.* (2010) and Giffin *et al.* (2010).

### Experimental

1,3-Bis(2,6-di-isopropylphenyl)1*H*-imidazol-3-ium chloride chloroform disolvate was prepared by reacting 0.05 g of 1,3-bis(2,6-diisopropylphenyl)-1,3-dihydro-2*H*-imidazol-2-ylidene with 0.05 ml of SiCl_4_ in deuterated dichloromethane. After two weeks at 253 K colorless needles of the title compound crystallized in the NMR-Tube.

### Refinement

H atoms were refined using a riding model, with C—H ranging from 0.95 Å to 1.00 Å and with *U*_iso_(H) = 1.2*U*_eq_(C) or *U*_iso_(H) = 1.5*U*_eq_(C_methyl_). The C—Cl distances of the dichloromethane molecules were restrained to be equal within an effective e.s.d. of 0.02 Å.

The highest maximum (1.34 e/Å^3^) in the final difference map is at 0.82 Å from Cl41 and the deepest hole (-1.59 e/Å^3^) is at 0.40 Å from Cl41.

### Crystal data

**Table:**

C_27_H_37_N_2_^+^·Cl^−^·2CH_2_Cl_2_	*F*(000) = 628
*M**_r_* = 594.89	*D*_x_ = 1.230 Mg m^−^^3^
Monoclinic, *P*2_1_/*m*	Mo *K*α radiation, λ = 0.71073 Å
Hall symbol: -P 2yb	Cell parameters from 27732 reflections
*a* = 9.1117 (4) Å	θ = 3.3–28.0°
*b* = 16.4990 (8) Å	µ = 0.47 mm^−^^1^
*c* = 10.8875 (6) Å	*T* = 173 K
β = 101.068 (4)°	Plate, colourless
*V* = 1606.32 (14) Å^3^	0.32 × 0.29 × 0.14 mm
*Z* = 2	

### Data collection

**Table:**

Stoe IPDS II two-circle diffractometer	2934 independent reflections
Radiation source: fine-focus sealed tube	2648 reflections with *I* > 2σ(*I*)
Graphite monochromator	*R*_int_ = 0.067
ω scans	θ_max_ = 25.0°, θ_min_ = 3.2°
Absorption correction: multi-scan (*MULABS*; Spek, 2009; Blessing, 1995)	*h* = −10→10
*T*_min_ = 0.864, *T*_max_ = 0.937	*k* = −19→19
17793 measured reflections	*l* = −12→12

### Refinement

**Table:**

Refinement on *F*^2^	Primary atom site location: structure-invariant direct methods
Least-squares matrix: full	Secondary atom site location: difference Fourier map
*R*[*F*^2^ > 2σ(*F*^2^)] = 0.075	Hydrogen site location: inferred from neighbouring sites
*wR*(*F*^2^) = 0.184	H-atom parameters constrained
*S* = 1.04	*w* = 1/[σ^2^(*F*_o_^2^) + (0.0696*P*)^2^ + 3.4645*P*] where *P* = (*F*_o_^2^ + 2*F*_c_^2^)/3
2934 reflections	(Δ/σ)_max_ < 0.001
185 parameters	Δρ_max_ = 1.36 e Å^−^^3^
6 restraints	Δρ_min_ = −1.60 e Å^−^^3^

### Special details

**Table:**

Geometry. All e.s.d.'s (except the e.s.d. in the dihedral angle between two l.s. planes) are estimated using the full covariance matrix. The cell e.s.d.'s are taken into account individually in the estimation of e.s.d.'s in distances, angles and torsion angles; correlations between e.s.d.'s in cell parameters are only used when they are defined by crystal symmetry. An approximate (isotropic) treatment of cell e.s.d.'s is used for estimating e.s.d.'s involving l.s. planes.
Refinement. Refinement of *F*^2^ against ALL reflections. The weighted *R*-factor *wR* and goodness of fit *S* are based on *F*^2^, conventional *R*-factors *R* are based on *F*, with *F* set to zero for negative *F*^2^. The threshold expression of *F*^2^ > σ(*F*^2^) is used only for calculating *R*-factors(gt) *etc*. and is not relevant to the choice of reflections for refinement. *R*-factors based on *F*^2^ are statistically about twice as large as those based on *F*, and *R*- factors based on ALL data will be even larger.

### Fractional atomic coordinates and isotropic or equivalent isotropic displacement parameters (Å^2^)

**Table:**

	*x*	*y*	*z*	*U*_iso_*/*U*_eq_	Occ. (<1)
Cl1	0.66661 (12)	0.2500	0.57563 (10)	0.0339 (3)	
N1	0.2042 (3)	0.31549 (14)	0.5835 (2)	0.0248 (5)	
C1	0.2891 (4)	0.2500	0.5788 (3)	0.0245 (8)	
H1	0.3918	0.2500	0.5730	0.029*	
C2	0.0606 (3)	0.29075 (18)	0.5907 (3)	0.0289 (6)	
H2	−0.0222	0.3250	0.5948	0.035*	
C11	0.2556 (3)	0.39874 (17)	0.5807 (3)	0.0298 (6)	
C12	0.2225 (4)	0.44066 (19)	0.4672 (3)	0.0356 (7)	
C13	0.2757 (4)	0.5201 (2)	0.4671 (3)	0.0484 (9)	
H13	0.2547	0.5510	0.3921	0.058*	
C14	0.3582 (5)	0.5546 (2)	0.5738 (4)	0.0571 (10)	
H14	0.3955	0.6081	0.5711	0.068*	
C15	0.3866 (5)	0.5115 (2)	0.6843 (4)	0.0503 (9)	
H15	0.4419	0.5365	0.7574	0.060*	
C16	0.3359 (4)	0.43202 (18)	0.6913 (3)	0.0358 (7)	
C21	0.1357 (4)	0.4026 (2)	0.3483 (3)	0.0386 (7)	
H21	0.1064	0.3468	0.3703	0.046*	
C22	0.2339 (9)	0.3934 (6)	0.2452 (8)	0.054 (2)*	0.50
H22A	0.3048	0.3487	0.2676	0.081*	0.50
H22B	0.2890	0.4438	0.2393	0.081*	0.50
H22C	0.1691	0.3820	0.1643	0.081*	0.50
C23	−0.0049 (9)	0.4473 (5)	0.2962 (8)	0.0508 (18)*	0.50
H23A	0.0003	0.5021	0.3314	0.076*	0.50
H23B	−0.0905	0.4185	0.3180	0.076*	0.50
H23C	−0.0169	0.4505	0.2049	0.076*	0.50
C22'	0.2392 (8)	0.3593 (5)	0.2818 (7)	0.0421 (16)*	0.50
H22D	0.1995	0.3606	0.1916	0.063*	0.50
H22E	0.2495	0.3029	0.3105	0.063*	0.50
H22F	0.3373	0.3858	0.2994	0.063*	0.50
C23'	0.0432 (10)	0.4686 (5)	0.2642 (8)	0.055 (2)*	0.50
H23D	−0.0477	0.4442	0.2157	0.082*	0.50
H23E	0.1035	0.4912	0.2069	0.082*	0.50
H23F	0.0159	0.5120	0.3171	0.082*	0.50
C61	0.3705 (4)	0.3863 (2)	0.8146 (3)	0.0405 (8)	
H61	0.3160	0.3334	0.8034	0.049*	
C62	0.5383 (5)	0.3685 (3)	0.8486 (3)	0.0561 (10)	
H62A	0.5600	0.3388	0.9281	0.084*	
H62B	0.5941	0.4196	0.8571	0.084*	
H62C	0.5680	0.3356	0.7826	0.084*	
C63	0.3195 (4)	0.4323 (2)	0.9205 (3)	0.0484 (9)	
H63A	0.3446	0.4008	0.9981	0.073*	
H63B	0.2111	0.4406	0.8995	0.073*	
H63C	0.3701	0.4849	0.9321	0.073*	
C3	0.8708 (8)	0.2675 (6)	0.8843 (7)	0.065 (4)	0.50
H3A	0.8224	0.2614	0.7953	0.078*	0.50
H3B	0.7940	0.2572	0.9355	0.078*	0.50
Cl31	0.9337 (5)	0.3663 (3)	0.9093 (4)	0.1291 (19)	0.50
Cl32	1.0117 (5)	0.1947 (4)	0.9208 (3)	0.149 (2)	0.50
C4	0.7100 (8)	0.2771 (5)	1.2652 (7)	0.059 (2)	0.50
H4A	0.7124	0.3367	1.2759	0.070*	0.50
H4B	0.7219	0.2526	1.3495	0.070*	0.50
Cl41	0.8547 (2)	0.2500	1.20316 (19)	0.1263 (12)	
Cl42	0.53459 (19)	0.2500	1.18007 (18)	0.0806 (6)	

### Atomic displacement parameters (Å^2^)

**Table:**

	*U*^11^	*U*^22^	*U*^33^	*U*^12^	*U*^13^	*U*^23^
Cl1	0.0375 (6)	0.0356 (6)	0.0296 (5)	0.000	0.0092 (4)	0.000
N1	0.0280 (12)	0.0222 (11)	0.0227 (11)	0.0004 (9)	0.0016 (9)	0.0000 (9)
C1	0.027 (2)	0.0238 (19)	0.0214 (18)	0.000	0.0013 (15)	0.000
C2	0.0276 (14)	0.0323 (14)	0.0268 (14)	0.0045 (12)	0.0057 (11)	0.0000 (12)
C11	0.0357 (15)	0.0213 (14)	0.0319 (15)	−0.0007 (12)	0.0049 (12)	0.0020 (11)
C12	0.0413 (17)	0.0313 (16)	0.0339 (16)	0.0033 (13)	0.0068 (13)	0.0055 (13)
C13	0.065 (2)	0.0337 (18)	0.046 (2)	−0.0017 (17)	0.0090 (17)	0.0153 (15)
C14	0.078 (3)	0.0266 (17)	0.064 (2)	−0.0151 (18)	0.007 (2)	0.0070 (17)
C15	0.067 (2)	0.0305 (17)	0.048 (2)	−0.0128 (17)	−0.0005 (18)	−0.0024 (15)
C16	0.0461 (18)	0.0249 (15)	0.0341 (16)	−0.0035 (13)	0.0021 (13)	−0.0012 (12)
C21	0.0444 (18)	0.0400 (18)	0.0304 (16)	0.0031 (14)	0.0048 (13)	0.0074 (13)
C61	0.057 (2)	0.0294 (16)	0.0303 (16)	−0.0070 (15)	−0.0027 (14)	−0.0027 (13)
C62	0.071 (3)	0.058 (2)	0.0366 (19)	0.023 (2)	0.0040 (17)	−0.0019 (17)
C63	0.050 (2)	0.054 (2)	0.0386 (18)	−0.0005 (17)	0.0038 (15)	−0.0060 (16)
C3	0.046 (4)	0.102 (14)	0.044 (3)	0.020 (5)	−0.001 (3)	0.011 (5)
Cl31	0.089 (3)	0.175 (5)	0.109 (3)	−0.071 (3)	−0.017 (2)	0.025 (3)
Cl32	0.116 (3)	0.282 (7)	0.0525 (16)	0.131 (4)	0.0274 (17)	0.040 (2)
C4	0.073 (5)	0.061 (6)	0.051 (4)	−0.013 (4)	0.034 (4)	−0.012 (3)
Cl41	0.0594 (11)	0.259 (4)	0.0599 (11)	0.000	0.0088 (9)	0.000
Cl42	0.0583 (10)	0.1116 (15)	0.0758 (11)	0.000	0.0227 (8)	0.000

### Geometric parameters (Å, º)

**Table:**

N1—C1	1.335 (3)	C22'—H22D	0.9800
N1—C2	1.388 (4)	C22'—H22E	0.9800
N1—C11	1.453 (4)	C22'—H22F	0.9800
C1—N1^i^	1.335 (3)	C23'—H23D	0.9800
C1—H1	0.9500	C23'—H23E	0.9800
C2—C2^i^	1.345 (6)	C23'—H23F	0.9800
C2—H2	0.9500	C61—C63	1.525 (5)
C11—C16	1.395 (4)	C61—C62	1.531 (5)
C11—C12	1.398 (4)	C61—H61	1.0000
C12—C13	1.398 (5)	C62—H62A	0.9800
C12—C21	1.517 (4)	C62—H62B	0.9800
C13—C14	1.379 (5)	C62—H62C	0.9800
C13—H13	0.9500	C63—H63A	0.9800
C14—C15	1.378 (5)	C63—H63B	0.9800
C14—H14	0.9500	C63—H63C	0.9800
C15—C16	1.398 (5)	C3—Cl31	1.732 (11)
C15—H15	0.9500	C3—Cl32	1.748 (9)
C16—C61	1.519 (4)	C3—H3A	0.9900
C21—C22'	1.479 (8)	C3—H3B	0.9900
C21—C23	1.493 (8)	Cl31—Cl32^i^	1.225 (7)
C21—C23'	1.562 (9)	Cl32—Cl31^i^	1.225 (7)
C21—C22	1.572 (9)	Cl32—C3^i^	1.414 (9)
C21—H21	1.0000	Cl32—Cl32^i^	1.825 (13)
C22—H22A	0.9800	C4—Cl41	1.654 (7)
C22—H22B	0.9800	C4—Cl42	1.744 (7)
C22—H22C	0.9800	C4—H4A	0.9900
C23—H23A	0.9800	C4—H4B	0.9900
C23—H23B	0.9800	Cl41—C4^i^	1.654 (7)
C23—H23C	0.9800	Cl42—C4^i^	1.744 (7)
			
C1—N1—C2	108.9 (2)	C21—C23—H23C	109.5
C1—N1—C11	124.9 (2)	H23A—C23—H23C	109.5
C2—N1—C11	126.2 (2)	H23B—C23—H23C	109.5
N1^i^—C1—N1	108.0 (3)	C21—C22'—H22D	109.5
N1^i^—C1—H1	126.0	C21—C22'—H22E	109.5
N1—C1—H1	126.0	H22D—C22'—H22E	109.5
C2^i^—C2—N1	107.11 (15)	C21—C22'—H22F	109.5
C2^i^—C2—H2	126.4	H22D—C22'—H22F	109.5
N1—C2—H2	126.4	H22E—C22'—H22F	109.5
C16—C11—C12	123.8 (3)	C21—C23'—H23D	109.5
C16—C11—N1	118.1 (3)	C21—C23'—H23E	109.5
C12—C11—N1	118.1 (3)	H23D—C23'—H23E	109.5
C11—C12—C13	116.7 (3)	C21—C23'—H23F	109.5
C11—C12—C21	122.6 (3)	H23D—C23'—H23F	109.5
C13—C12—C21	120.8 (3)	H23E—C23'—H23F	109.5
C14—C13—C12	121.2 (3)	C16—C61—C63	112.3 (3)
C14—C13—H13	119.4	C16—C61—C62	109.7 (3)
C12—C13—H13	119.4	C63—C61—C62	110.4 (3)
C15—C14—C13	120.3 (3)	C16—C61—H61	108.1
C15—C14—H14	119.9	C63—C61—H61	108.1
C13—C14—H14	119.9	C62—C61—H61	108.1
C14—C15—C16	121.5 (3)	C61—C62—H62A	109.5
C14—C15—H15	119.3	C61—C62—H62B	109.5
C16—C15—H15	119.3	H62A—C62—H62B	109.5
C11—C16—C15	116.5 (3)	C61—C62—H62C	109.5
C11—C16—C61	123.6 (3)	H62A—C62—H62C	109.5
C15—C16—C61	119.9 (3)	H62B—C62—H62C	109.5
C22'—C21—C23	129.3 (5)	C61—C63—H63A	109.5
C22'—C21—C12	109.9 (4)	C61—C63—H63B	109.5
C23—C21—C12	112.9 (4)	H63A—C63—H63B	109.5
C22'—C21—C23'	111.9 (5)	C61—C63—H63C	109.5
C12—C21—C23'	110.2 (4)	H63A—C63—H63C	109.5
C23—C21—C22	109.9 (5)	H63B—C63—H63C	109.5
C12—C21—C22	112.0 (4)	Cl31—C3—Cl32	113.9 (5)
C23'—C21—C22	88.0 (5)	Cl31—C3—H3A	108.8
C22'—C21—H21	84.1	Cl32—C3—H3A	108.8
C23—C21—H21	107.2	Cl31—C3—H3B	108.8
C12—C21—H21	107.2	Cl32—C3—H3B	108.8
C23'—C21—H21	130.0	H3A—C3—H3B	107.7
C22—C21—H21	107.2	Cl32^i^—Cl31—C3	53.9 (4)
C21—C22—H22A	109.5	Cl31^i^—Cl32—C3^i^	81.7 (5)
C21—C22—H22B	109.5	Cl31^i^—Cl32—C3	98.9 (5)
H22A—C22—H22B	109.5	Cl31^i^—Cl32—Cl32^i^	145.3 (3)
C21—C22—H22C	109.5	Cl41—C4—Cl42	115.8 (4)
H22A—C22—H22C	109.5	Cl41—C4—H4A	108.3
H22B—C22—H22C	109.5	Cl42—C4—H4A	108.3
C21—C23—H23A	109.5	Cl41—C4—H4B	108.3
C21—C23—H23B	109.5	Cl42—C4—H4B	108.3
H23A—C23—H23B	109.5	H4A—C4—H4B	107.4
			
C2—N1—C1—N1^i^	−0.4 (4)	C14—C15—C16—C11	0.1 (6)
C11—N1—C1—N1^i^	179.95 (19)	C14—C15—C16—C61	179.5 (4)
C1—N1—C2—C2^i^	0.3 (2)	C11—C12—C21—C22'	−87.2 (5)
C11—N1—C2—C2^i^	179.9 (2)	C13—C12—C21—C22'	91.7 (5)
C1—N1—C11—C16	−79.7 (4)	C11—C12—C21—C23	120.6 (5)
C2—N1—C11—C16	100.8 (3)	C13—C12—C21—C23	−60.5 (6)
C1—N1—C11—C12	100.0 (4)	C11—C12—C21—C23'	149.1 (5)
C2—N1—C11—C12	−79.6 (4)	C13—C12—C21—C23'	−32.1 (5)
C16—C11—C12—C13	0.7 (5)	C11—C12—C21—C22	−114.7 (5)
N1—C11—C12—C13	−178.9 (3)	C13—C12—C21—C22	64.2 (5)
C16—C11—C12—C21	179.6 (3)	C11—C16—C61—C63	−125.9 (3)
N1—C11—C12—C21	0.0 (4)	C15—C16—C61—C63	54.7 (5)
C11—C12—C13—C14	0.7 (5)	C11—C16—C61—C62	110.9 (4)
C21—C12—C13—C14	−178.2 (4)	C15—C16—C61—C62	−68.4 (4)
C12—C13—C14—C15	−1.7 (7)	Cl32—C3—Cl31—Cl32^i^	4.6 (3)
C13—C14—C15—C16	1.2 (7)	Cl31—C3—Cl32—Cl31^i^	172.1 (6)
C12—C11—C16—C15	−1.1 (5)	Cl31—C3—Cl32—C3^i^	176.6 (3)
N1—C11—C16—C15	178.5 (3)	Cl31—C3—Cl32—Cl32^i^	−3.4 (3)
C12—C11—C16—C61	179.5 (3)	Cl42—C4—Cl41—C4^i^	−64.5 (5)
N1—C11—C16—C61	−0.9 (5)	Cl41—C4—Cl42—C4^i^	64.0 (5)

Symmetry code: (i) *x*, −*y*+1/2, *z*.

### Hydrogen-bond geometry (Å, º)

**Table:**

*D*—H···*A*	*D*—H	H···*A*	*D*···*A*	*D*—H···*A*
C1—H1···Cl1	0.95	2.50	3.447 (4)	176

## References

[bb1] Arduengo, A. J., Goerlich, J. R. & Marshall, W. J. (1995). *J. Am. Chem. Soc.* **117**, 11027–11028.

[bb2] Arduengo, A. J., Krafczyk, R., Schmutzler, R., Craig, H. A., Goerlich, J. R., Marshall, W. J. & Unverzagt, M. (1999). *Tetrahedron*, **55**, 14523–14534.

[bb3] Berger, M., Auner, N., Sinke, T. & Bolte, M. (2012). *Acta Cryst.* E**68**, o1845. 10.1107/S1600536812022246 PMC3379414 22719612

[bb4] Blessing, R. H. (1995). *Acta Cryst.* A**51**, 33–38. 10.1107/s0108767394005726 7702794

[bb5] Blue, E. D., Gunnoe, T. B., Petersen, J. L. & Boyle, P. D. (2006). *J. Organomet. Chem.* **691**, 5988–5993.

[bb6] Giffin, N. A., Hendsbee, A. D. & Masuda, J. D. (2010). *Acta Cryst.* E**66**, o2090–o2091. 10.1107/S1600536810028424 PMC3007462 21588386

[bb7] Hintermann, L. (2007). *Beilstein J. Org. Chem.* **3** No. 22. doi:10.1186/1860-5397-3-22. 10.1186/1860-5397-3-22 PMC2151073 17725838

[bb8] Ikhile, M. I. & Bala, M. D. (2010). *Acta Cryst.* E**66**, o3121. 10.1107/S1600536810045228 PMC3011707 21589425

[bb9] Sheldrick, G. M. (2008). *Acta Cryst.* A**64**, 112–122. 10.1107/S0108767307043930 18156677

[bb10] Spek, A. L. (2009). *Acta Cryst.* D**65**, 148–155. 10.1107/S090744490804362X PMC2631630 19171970

[bb11] Stasch, A., Singh, S., Roesky, H. W., Noltemeyer, M. & Schmidt, H.-G. (2004). *Eur. J. Inorg. Chem.* pp. 4052–4055.

[bb12] Stoe & Cie (2001). *X-AREA* Stoe & Cie, Darmstadt, Germany.

